# ^177^Lu-PSMA Radioligand Therapy Is Favorable as Third-Line Treatment of Patients with Metastatic Castration-Resistant Prostate Cancer. A Systematic Review and Network Meta-Analysis of Randomized Controlled Trials

**DOI:** 10.3390/biomedicines9081042

**Published:** 2021-08-19

**Authors:** Finn E. von Eyben, Kalevi Kairemo, Channing Paller, Manuela Andrea Hoffmann, Giovanni Paganelli, Irene Virgolini, Giandomenico Roviello

**Affiliations:** 1 Center for Tobacco Control Research, Birkevej 17, DK-5230 Odense M, Denmark; 2 Docrates Cancer Center, Saukanpaaderanta 2, 18000 Helsinki, Finland; kalevi.kairemo@gmail.com; 3 Department of Nuclear Medicine, The University of Texas MD Anderson Cancer Center, 1515 Holcombe Boulevard, Houston, TX 77030, USA; 4 Sidney Kimmel Comprehensive Cancer Center, John Hopkins University School of Medicine, 3400 N. Charles Street, Baltimore, MD 21218, USA; cpaller1@jhmi.edu; 5 Department of Occupational Health & Safety, Federal Ministry of Defense, Fontaingraben 150, 53123 Bonn, Germany; manuhoffmann@web.de; 6 Department of Nuclear Medicine, University Medical Center of the Johannes Guttenberg University in Mainz, Langenbeckerstrasse 15, 55101 Mainz, Germany; 7 Istituto Scientifico Romagnolo per lo Studio e la Cura Tumori, IRST, Via Piero Maroncelli, 4704 Meldola, Italy; giovanni.paganelli@irst.emr.it; 8 Department of Nuclear Medicine, University Hospital in Innsbruck, Wilhelm-Geil Strasse 25, 6020 Innsbruck, Austria; irene.virgolini@i-med.ac.at; 9 Department of Health Sciences, Section of Clinical Pharmacology and Oncology, University of Florence, Piazza S. Marco 4, 50121 Florence, Italy; giandomenicoroviello@gmail.com

**Keywords:** advanced metastatic castration-resistant prostate cancer, connected network, frequentist analysis, benefits and harms of treatments, ranking of treatments

## Abstract

In this systematic review and network meta-analysis (NMA), we aimed to assess the benefits and harms of third-line (L3) treatments in randomized controlled trials (RCTs) of patients with metastatic castration-resistant prostate cancer (mCRPC). Two reviewers searched for publications from 1 January 2006 to 30 June 2021. The review analyzed seven RCTs that included 3958 patients and eight treatments. Treatment with prostate-specific membrane antigen (PSMA)-based radioligand therapy (PRLT) resulted in a 1.3-times-higher rate of median PSA decline ≥50% than treatment with abiraterone, enzalutamide, mitoxantrone, or cabazitaxel (*p* = 0.00001). The likelihood was 97.6% for PRLT to bring about the best PSA response, out of the examined treatments. PRLT resulted in a 1.1-times-higher six-month rate of median radiographic progression-free survival. Treatment with PRLT in the VISION trial resulted in 1.05-times-higher twelve-month median overall survival than L3 treatment with cabazitaxel in other RCTs. PRLT more often resulted in severe thrombocytopenia and less often in severe leukopenia than did cabazitaxel. In conclusion, for patients with mCRPC, L3 treatment with PRLT is highly effective and safe.

## 1. Introduction

For men in Western societies, prostate cancer is the second leading cause of cancer death [1]. Most deaths from prostate cancer are due to advanced metastatic castration-resistant prostate cancer (mCRPC). Several drugs prolong life for patients with mCRPC [2]. In 2018, guidelines from the European Association of Urology (EAU) recommended that, as a first-line (L1) treatment, patients with mCRPC be treated either with a combination of androgen deprivation therapy (ADT) and abiraterone or with a combination of ADT and docetaxel [3].

Established drugs prolong median overall survival by many months [4,5]. In routine practice, half of the patients who progress on L1 treatment are treated with a second line (L2) treatment, and half of the patients progressing on L2 treatment are treated with a third line (L3) treatment [6,7]. Thus, in the European Union each year, more than 20,000 patients may be candidates for L3 treatment.

L1 treatment nowadays often combines ADT and an androgen receptor pathway inhibitor (ARPI) [8], and L2 treatment often consists of docetaxel [6], but some centers still use chemotherapy as an L1 treatment and ARPI as an L2 treatment. During recent years, randomized controlled trials (RCTs) explored L3 treatment with abiraterone, enzalutamide, and cabazitaxel [9]. L3 treatment often consists of cabazitaxel, and clinical characteristics indicate the patients most likely to respond [7].

A systematic review and meta-analysis in 2018 summarized the outcomes and effects of established drugs used as L3 treatments [10]. Surprisingly, patients with end-stage prostate cancer treated with ^177^Lu-PSMA-based radioligand therapy (PRLT) showed better PSA responses than patients with mCRPC did after the L3 treatments. Other systematic reviews also evaluated treatment with PRLT [1,2,3,4,5,6,11,12,13,14,15,16,17,18].

Correspondingly, in a recent RCT, the TheraP trial, L3 treatment with PRLT yielded a better PSA response and better long-term radiographic progression-free survival than L3 treatments with cabazitaxel [19]. PRLT prolonged median overall survival compared with best supportive care (BSC) in another recent RCT, the VISION trial [20]. However, the TheraP and VISION trials compared PRLT with two L3 treatments, whereas other RCTs evaluated eight treatments.

Therefore, we carried out a network meta-analysis (NMA) of RCTs of L3 treatments with the aim of analyzing the clinical benefits and harms and the relative efficacy of the eight treatments. Our systematic review followed the Participant, Intervention, Comparator, and Outcome (PICO) guidelines.

Participants (P) were patients with histologically proven prostate cancer, metastases, and serum testosterone levels <0.5 ng/dL (<1.73 nmol/L) (mCRPC), who had progressed on or shown intolerance to (1) two ADT treatments and (2) docetaxel, and (3) who had participated in an RCT of L3 treatment. Interventions (I) were the L3 treatments. The comparator L3 treatment, C, was cabazitaxel administered in the dosage of 25 mg/m^2^ body surface.

The RCTs were terminated when they reached 70% to 85% of the planned events. We chose overall survival as the primary effect outcome (O) at the start of the NMA in February 2021. We also evaluated the best PSA response and radiological progression-free survival, as recommended by the Prostate Cancer Clinical Trial Working Group 3 (PCWG3) [21]. As for clinical harm, we evaluated the rates of deaths due to severe adverse effects, the rates of severe hematologic adverse effects, and the rates of premature discontinuation of treatment due to adverse effects.

## 2. Material and Methods

### 2.1. Selection of Studies

The systematic review in our NMA followed the Preferred Reporting Items for Systematic Reviews and Meta-analysis for Network Meta-analysis (PRISMA-NMA) guidelines [22,23,24]. The review included only RCTs published after 2005 in order to assure that the evaluations of the treatments had a high quality, and to focus on the recent developments of effective drugs for patients with mCRPC. Two reviewers (F.E.v.E. and G.R.) searched for publications from 1 January 2006 to 30 June 2021. A third reviewer (G.P.) made a final decision if the two reviewers disagreed.

**Figure 1:**
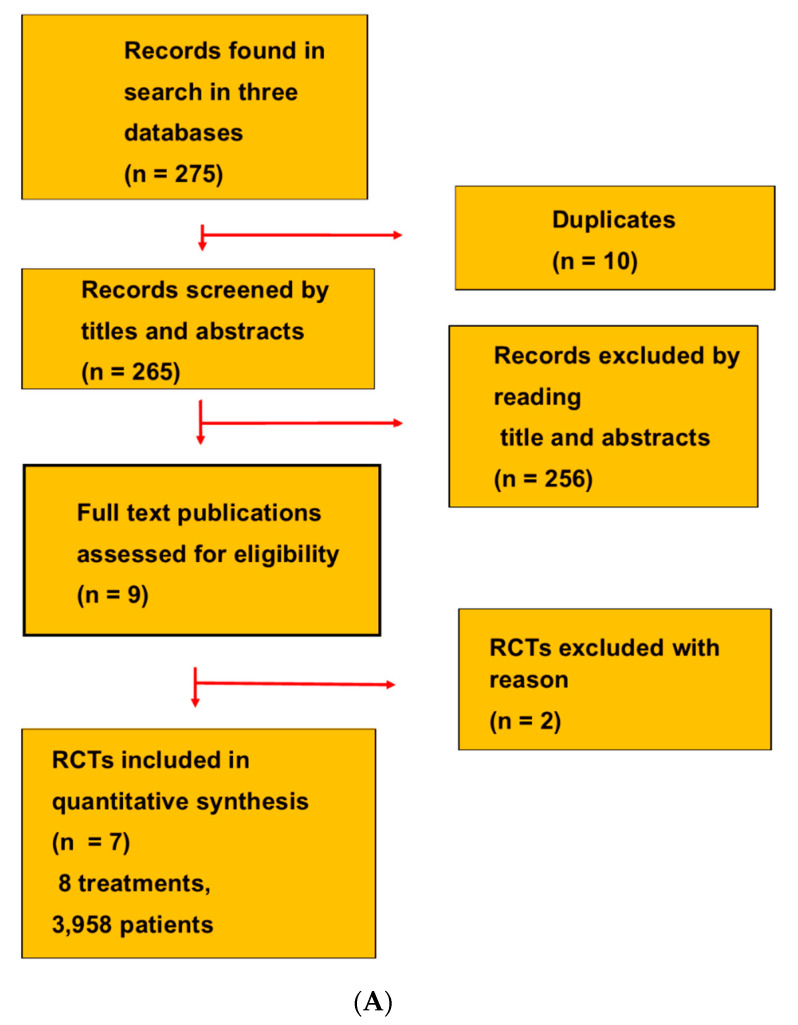

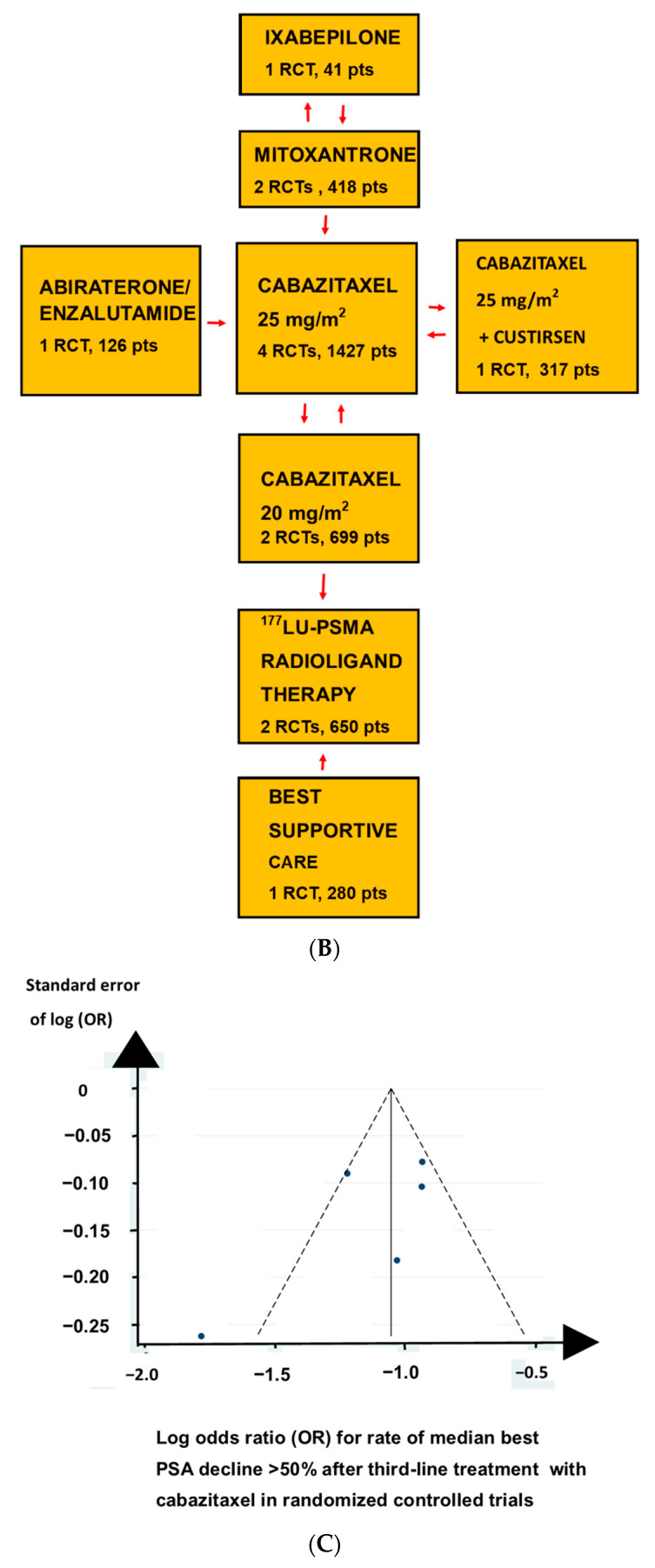
(**A**). In this systematic review of the network meta-analysis, we searched for RCTs in three databases. The selection process is shown as a PRISMA flow diagram. (**B**). The L3 treatments connected the RCTs in a network with a ladder and a star configuration without any loops. A single arrow between two treatments points to the most effective treatment, and double arrows indicate non-inferiority between the treatments. (**C**). L3 treatment with cabazitaxel resulted in rates of median PSA decline ≥50% that had a symmetric distribution with only one outlier in a funnel plot.

The reviewers searched for literature in PubMed, Embase, and the Cochrane Central Register of Controlled Trials (Figure 1A). A manual search in reference lists was also undertaken in the publications of the RCTs and in reviews.

### 2.2. Search Strategy

For the search in Pubmed, the reviewers used the MESH words and text words (prostat* cancer* OR prostate adenocarcinoma OR prostat* neoplasm*) AND (castration resistance) AND (docetaxel resistance OR docetaxel failure) AND (randomized controlled trial*). The reviewers also searched in Embase and Cochrane Clinical Controlled Trials using similar search terms. The reviewers translated the search terms to similar search terms for the searches in other databases. The search produced 275 records.

The systematic review in our NMA excluded reviews; publications not written in the English language; publications on prostate cancers that are different from prostate adenocarcinoma; publications on RCTs of L1 and L2 treatments, such as the ALSYMPCA trial [25]; publications of single-center non-randomized cohort studies; and publications of laboratory investigations of the biology of prostate cancer.

The reviewers contacted the corresponding authors of the publications of the RCTs in regard to data missing from the publications, but the data were not available.

### 2.3. Data Extraction

The two reviewers independently extracted the clinical characteristics of patients who underwent the treatments reported in the RCTs. These characteristics were used to document the transitivity of the NMA.

The L3 treatments were (1) best supportive care: treatment without active anticancer drugs or other drugs with no known interaction with PRLT; (2) ixabepilone given as 35 mg/m^2^ body surface intravenously every three weeks; (3) mitoxantrone given as 14 mg/m^2^ intravenously every three weeks; (4) cabazitaxel given as monotherapy in the dosage of 25 mg/m^2^ body surface intravenously every three weeks; (5) combination therapy with cabazitaxel 25 mg/m^2^ + custirsen given with custirsen at 640 mg weekly; (6) cabazitaxel given as monotherapy in the dosage of 20 mg/m^2^ body surface intravenously every three weeks; (7) second-line alterative ARPIs, such as abiraterone given with 1000 mg a day or enzalutamide given with 160 mg a day; and (8) PRLT given with 7.4–8.4 GBq ^177^Lu intravenously every six weeks.

The reviewers also registered the duration of follow-up, fourth-line (L4) treatments, median best PSA decline ≥50%, radiographic progression-free survival, and overall survival. The reviewers also extracted data on severe adverse effects.

### 2.4. Definitions

Patients with mCRPC were defined as patients who had PSA recurrence after initial treatment, had progression despite treatment reducing testosterone to castration levels, and had metastatic sites detected with bone scans and CT scans [2].

PSMA-positive sites were defined as sites that had a higher tracer uptake on ^68^Ga-PSMA PET/CT scans than normal liver parenchyma, and PSMA-negative sites were defined as sites that had a lower tracer uptake on ^68^Ga-PSMA PET/CT than liver parenchyma. PSMA-positive sites were mandatory for patients in the PRLT trials.

Treatment of patients with mCRPC that was resistant to ≥2 series of ADT and to chemotherapy with docetaxel was defined as L3 treatment.

The reference treatment in the NMA was defined as cabazitaxel given as 25 mg/m^2^ body surface, which was the L3 treatment reported in most RCTs. PSA response was defined as the median best PSA decline ≥50% [21]. Radiological progression-free survival was defined as the time span from the start of L3 treatment to radiological progression, according to the Response Evaluation Criteria in Solid Tumors (RECIST) for bone scans and CT scans [26], evaluated as the proportion of patients examined at six months of follow-up.

Overall survival was defined as the time span from the start of the L3 treatment to the death of the patients or to the end of follow-up, evaluated as the proportion of patients surviving at twelve months of follow-up.

Severe adverse effects related to the L3 treatment were defined as grade 3 and 4 adverse effects according to the Common Terminology Criteria for Adverse Effects, version 3.0 or 4.0 (CTCAE v3.0 or 4.0). Rates of premature discontinuation of treatments due to adverse effects were defined as the rates reported in the publications of the RCTs. However, the TheraP trialists reported premature discontinuation of the treatments for several subgroups of patients.

The grade of the evidence for the treatments was defined as the grades, as determined according to the GRADE system [27].

### 2.5. Assessment of Risk of Bias

The design of the RCTs implied a risk for bias. Oncologists carried out the L3 treatments without being blinded for the type of treatments, and evaluators evaluated the outcomes without being blinded for the treatments. To evaluate the risk of significant bias in the RCTs, we carried out a funnel plot of the PSA response to L3 treatments with cabazitaxel.

### 2.6. Statistical Analyses

Data that were missing from the publications of the RCTs were not substituted. We used a frequentist approach and evaluated whether the RCTs had rather similar clinical characteristics and whether treatments examined in more than one RCT had similar effect outcomes. A random effects model was chosen, because RCTs in the recent decade increasingly included ARPIs as L1 and L2 treatments. We used a multivariate indirect model.

A DerSemonian and Laird model was used to evaluate heterogeneity between the RCTs [28,29]. Effect outcomes were two interim endpoints and the main endpoint [28,30,31,32]. Outcomes for a treatment in the RCTs, such as the median best PSA decline ≥50%, were summarized using the method of Nyaga et al. [33]. We selected a random effects model. The method showed the results as forest plots, irrespective of the size of the proportions for the outcome.

Progression-free survival and overall survival were analyzed based on the proportion of patients examined at three- and six-month intervals in the follow-up, adopted by summarizing the data reported in risk tables for Kaplan–Meier plots in the publications as forest plots. Treatment rankings were calculated, representing the rescaled mean rankings [30].

The ranking of treatments regarding the PSA response, radiographic progression-free survival, and overall survival was possible only for RCTs that were connected in a network of treatments. The ranking expressed the probability of a treatment to represent the best or the worst treatment regarding the outcomes. The calculations were carried out using a software package, “network” by I. R. White, for the statistical software program STATA [34]. This ranking may be used for the grading of the evidence for the treatments [35,36].

We also evaluated severe adverse effects. A *p*-value < 0.05 was considered to be statistically significant. All statistical analyses were carried out using STATA 17 (Stata corp. College Station, TX, USA).

## 3. Results

### 3.1. The Selected RCTs

The NMA selected seven RCTs that evaluated eight L3 treatments (Figure 1A, a PRISMA flow diagram, Table 1): the IXA, TROPIC, PROSELICA, AFFINITY, CARD, TheraP, and VISION trials [9,19,20,37,38,39,40]. The RCTs were two-armed multicenter trials. Five RCTs had a high quality and two had minor problems. The selected RCTs were published in leading medical journals after 2005. The treatments in the RCTs formed a connected network with a ladder and star configuration without loops between the treatments (Figure 1B).

The NMA included 3958 patients. The patients in the eight treatments of the RCTs had rather similar clinical characteristics (Table 1). The TROPIC, CARD, TheraP, and VISION trials had sufficient sizes and follow-ups to point out that a treatment was significantly better than another treatment. In contrast, the IXA, AFFINITY (hazard ratio 0.95), and PROSELICA trials (hazard ratio 1.02) were non-inferiority RCTs.

One RCT included <100 participants, two RCTs included 100–600 participants, and four RCTs included >600 participants. Five RCTs reported the effect outcomes evaluated in our NMA, whereas the PROSELICA trial did not report the PSA response [38], and the TheraP trial did not report overall survival [19]. Five of the eight treatments differed in efficacy in pairwise comparisons between the RCTs (Figure 1B).

Four RCTs included cabazitaxel treatment given as 25 mg/m^2^ body surface and two RCTs included cabazitaxel treatment given as 20 mg/m^2^ body surface. In two RCTs, PRLT was given as ^177^Lu-PSMA-617 with 7.4–8.4 GBq ^177^Lu in the cycles and with six-week intervals between the cycles [19,20]. Four treatments were investigated in at least two RCTs, whereas four other treatments were investigated in only a single RCT.

The PSA response after cabazitaxel in the RCTs did not reveal a major bias in a funnel plot (Figure 1C).

### 3.2. Clinical Benefits

**Figure 2:**
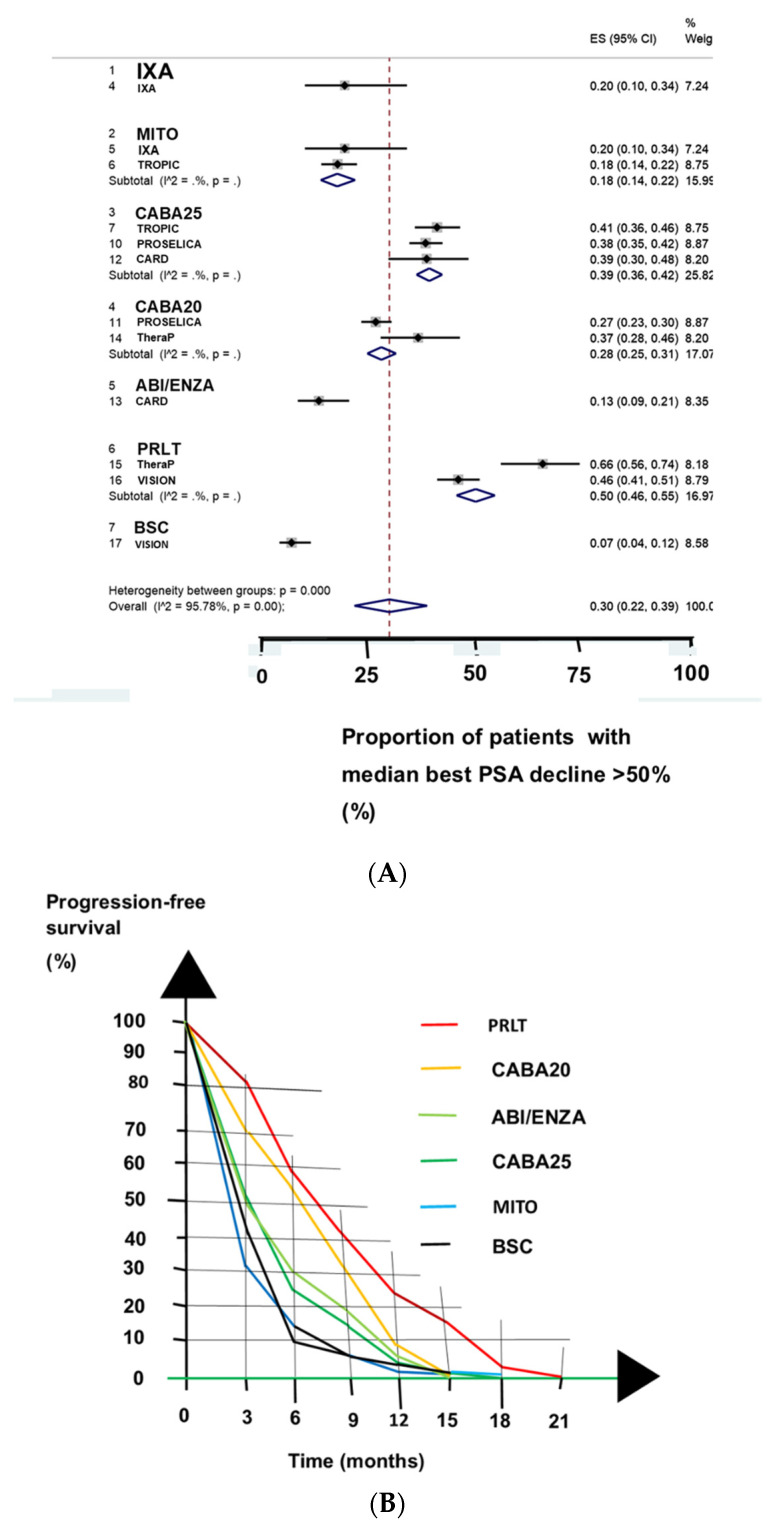

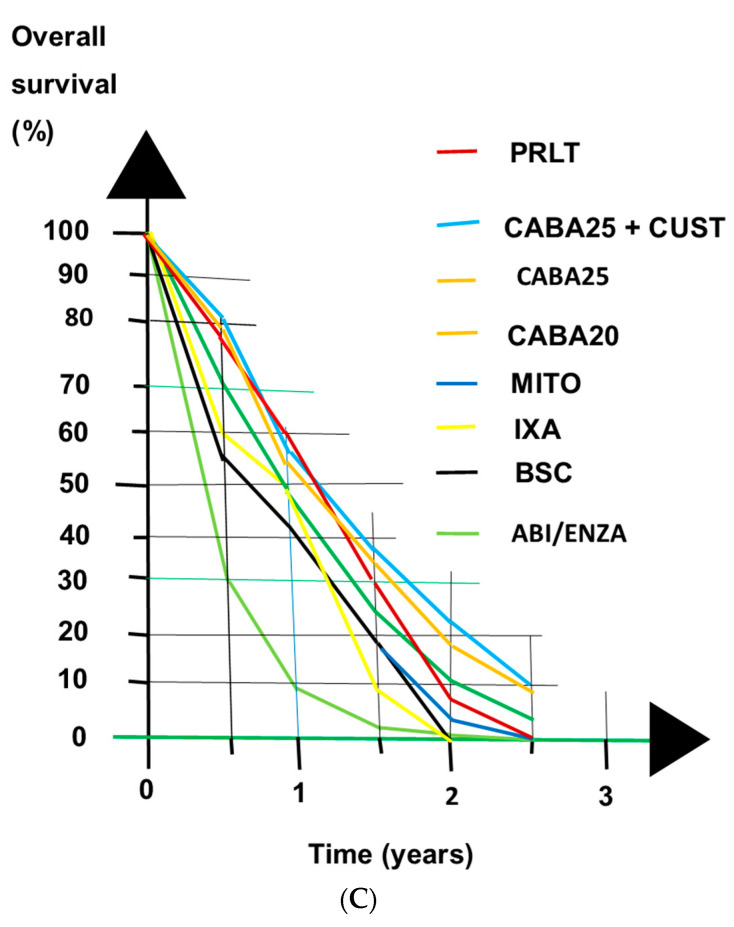
(**A**) PRLT resulted in the best median PSA decline ≥50% of the seven treatments evaluated, as shown in a forest plot. MITO denotes mitoxantrone, CABA25 denotes cabazitaxel given as 25 mg/m^2^ body surface, CABA20 denotes cabazitaxel given as 20 mg/m^2^ body surface, ABI/ENZA denotes treatment with the alternative ARPI of patients who had failed to abiraterone or enzalutamide, and PRLT denotes PSMA-based radioligand therapy. (**B**) PRLT resulted in the best radiographic progression-free survival of the five L3 treatments evaluated. The black line shows the best supportive care (BSC), the blue line shows treatment with mitoxantrone (MI), the dark-green line shows treatment with cabazitaxel given as 25 mg/m^2^ body surface (CABA25), the light-green line shows alternative treatment with abiraterone or enzalutamide (ABI/ENZA), the orange line shows treatment with cabazitaxel given as 20 mg/m^2^ body surface (CABA20), and the red line shows treatment with PRLT. (**C**) Only at 12 months of follow-up did L3 treatment with PRLT result in a slightly better overall survival than other treatments. The light-green line shows the treatment with the alternative abiraterone or enzalutamide, the black line shows best supportive care, the yellow line shows treatment with ixabepilone, the deep-blue line shows treatment with mitoxantrone, the orange line shows treatment with cabazitaxel 20 mg/m^2^ body surface, the deep-green line shows treatment with cabazitaxel 25 mg/m^2^ body surface, the light-blue line shows the combination treatment of cabazitaxel and custirsen, and the deep-red line shows treatment with PRLT.

PRLT resulted in a higher rate of median best PSA decline ≥50% than did cabazitaxel, abiraterone, enzalutamide, or mitoxantrone (*p* = 0.00001, Table 2 and Figure 2A). PRLT resulted in a 1.3-times-higher proportion of patients with of PSA response than cabazitaxel given as 25 mg/m^2^ body surface. We obtained a 97.6% probability that PRLT was the best among the eight treatments (Table 3).

Cabazitaxel given as 25 mg/m^2^ body surface resulted in a higher PSA response than cabazitaxel given as 20 mg/m^2^ body surface. An alternative ARPI approach and best supportive care were the two worst treatments in regard to the PSA response.

PRLT resulted in 1.1-times-higher proportion with radiographic progression-free survival at six months’ follow-up compared with cabazitaxel (Figure 2B). Cabazitaxel resulted in better survival than an alternative abiraterone or enzalutamide. PRLT had a high ranking as an L3 treatment in regard to radiographic progression-free survival (Table 4).

PRLT in the VISION trial resulted in a slightly longer median overall survival (15.3 months) than other treatments in the RCTs (Figure 2C) [13]. PRLT in the VISION trial resulted in a 1.05-times-higher proportion of patients with of overall survival at 12 months’ follow-up than cabazitaxel did in other RCTs. Patients who had failed to respond to abiraterone or enzalutamide and were treated with cabazitaxel lived longer than patients who were treated with an alternative ARPI.

Regarding the rankings for overall survival, combination therapy with cabazitaxel and custirsen was the best L3 treatment with a median overall survival of 14.1 months, and the alternative abiraterone or enzalutamide approaches were the worst L3 treatments (Table 5). However, this ranking analysis did not include the VISION trial and PRLT.

### 3.3. Adverse Effects

**Figure 3:**
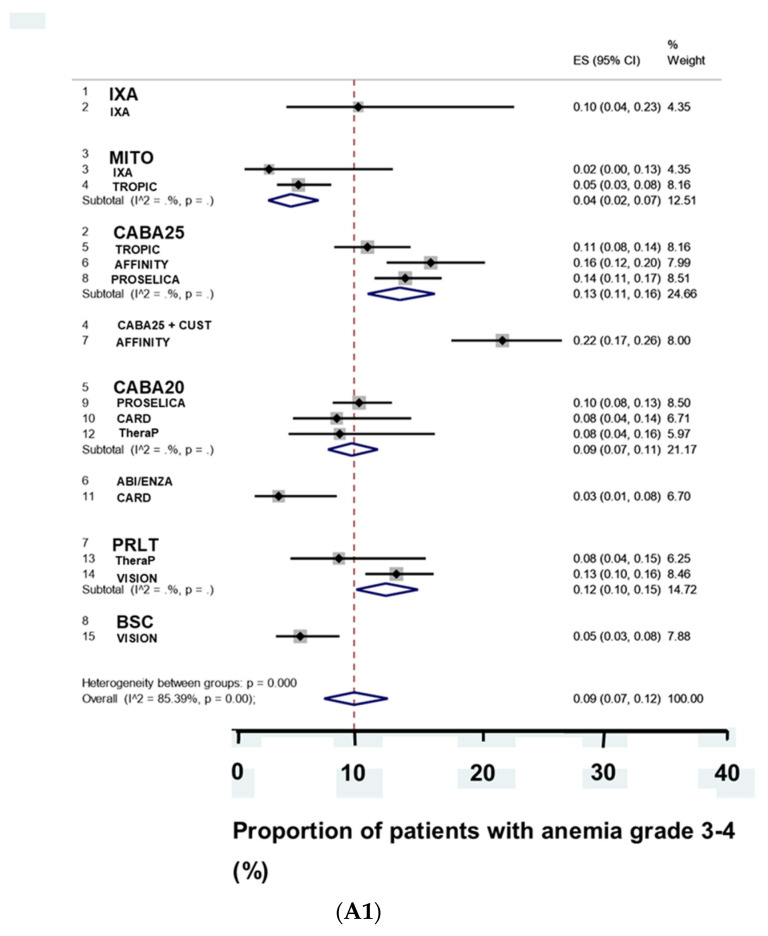

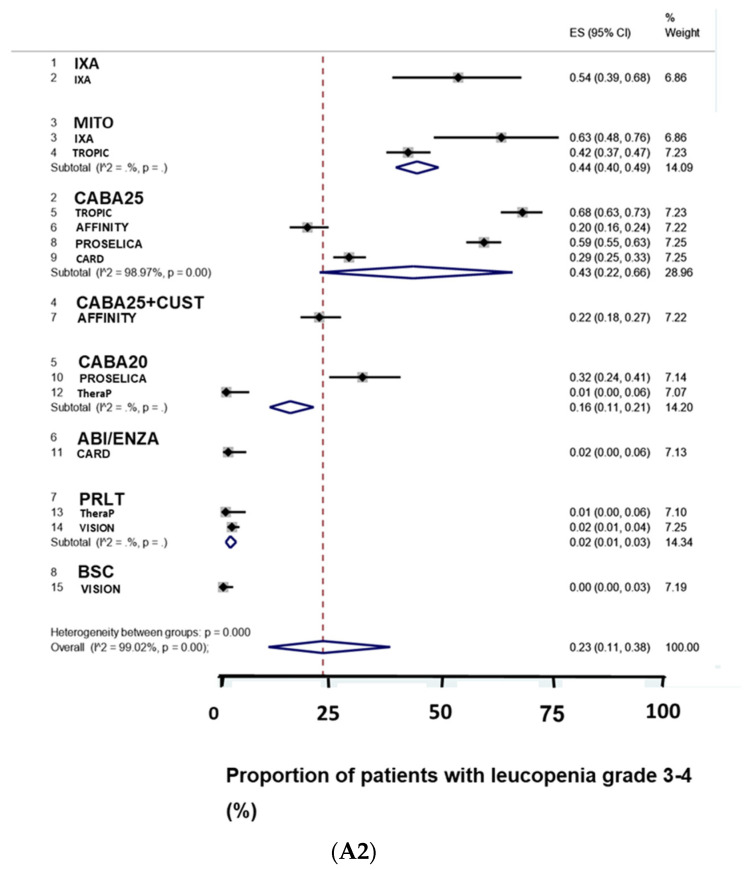

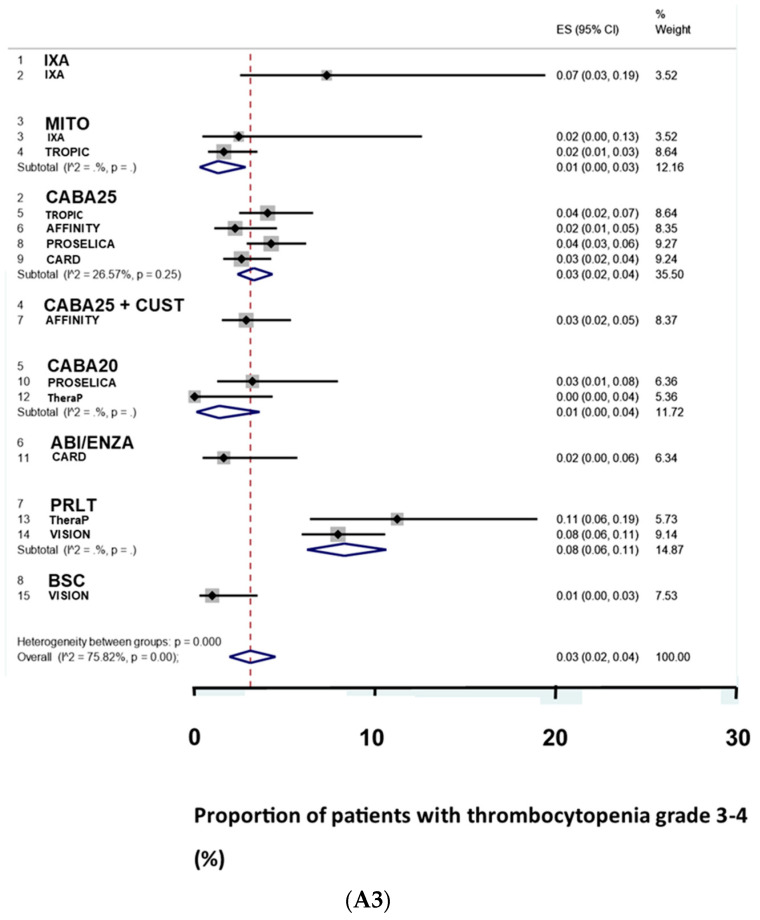

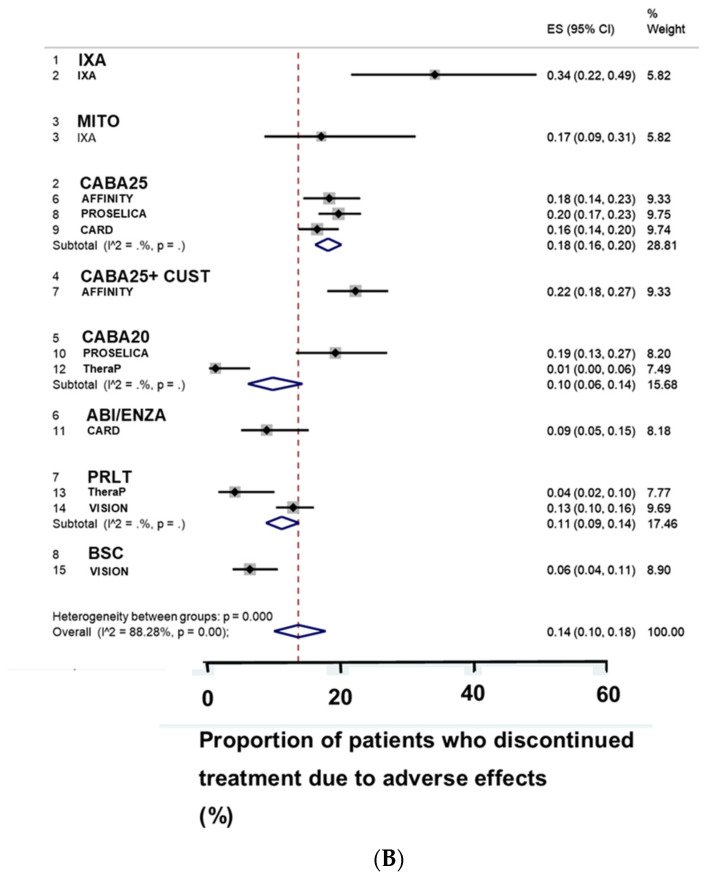
(**A1**). The proportion of patients with severe anemia was higher after L3 treatment with a combination of cabazitaxel and custirsen than after treatment with ixabepilone, cabazitaxel as monotherapy, and PRLT. The proportion of patients with severe anemia was lowest with alternative abiraterone or enzalutamide and best supportive care. (**A2**). The proportion of patients with severe leukopenia was higher after treatment with ixabepilone than after treatment with mitoxantrone, cabazitaxel 25 mg/m^2^ body surface, and the combination treatment of cabazitaxel and custirsen. The proportion of patients with severe leucopenia was lower after treatment with cabazitaxel, the alternative abiraterone and enzalutamide, and PRLT; (**A3**) the proportion of patients with severe thrombocytopenia was higher after treatment with the combination of cabazitaxel and custirsen than after treatment with cabazitaxel 25 mg/m^2^ body surface and PRLT. The proportion of patients with severe thrombocytopenia was lowest after treatment with mitoxantrone and best supportive care. (**B**) The proportion of patients with premature discontinuation of treatment due to severe adverse effects was <10% after the L3 treatments. The proportion of patients with discontinuation of treatment was highest after treatment with ixabepilone and PRLT.

A small percentage of the patients treated with L3 cabazitaxel died of severe adverse effects. Otherwise, the treatments resulted in modest rates of severe adverse effects. Cabazitaxel and PRLT did not differ significantly in regard to the proportion of patients with of severe anemia (Figure 3A1). PRLT resulted in less severe leukopenia and more severe thrombocytopenia than did cabazitaxel (Figure 3A2,A3).

The proportion of patients with premature discontinuation of treatment varied considerably between the L3 treatments (Figure 3B). Treatment with cabazitaxel as 25 mg/m^2^ body surface more often resulted in discontinued treatment due to adverse effects than did treatment with cabazitaxel as 20 mg/m^2^ body surface.

## 4. Discussion

The present NMA is the first NMA to include PRLT as one of the L3 treatments and the first NMA to designate PRLT as the preferred L3 treatment. The NMA confirmed the efficacy of PRLT, as previously reported in a meta-analysis regarding patients with end-stage prostate cancer [10]. Similarly, PRLT was found to result in a better PSA response than salvage radiotherapy and abiraterone in a previous case report [41].

PRLT had an important role for patients with advanced mCRPC both in the TheraP and the VISION trials [19,20], and in our NMA. The two RCTs provided grade A/B evidence that PRLT is an effective treatment. The VISION trial included a subgroup of patients who had been treated with cabazitaxel before they were treated with PRLT, so future analyses in the VISION trial of PRLT given as an L3 treatment might be expected to demonstrate a better overall survival than that reported so far for all patients in the trial. The NMA provides an indirect comparison between PRLT and six other L3 treatments.

Recommendations for L3 treatment based on our NMA may be applied for most patients with mCRPC in most circumstances, but future subgroup analyses of the TheraP and VISION trials may modify our results.

At the start of the NMA, we chose overall survival as the main outcome, and also evaluated two interim outcomes [31]. First, on 23 June 2021, results on overall survival after L3 treatment with PRLT were published [20]. Thus, we chose our main outcome before we knew the outcomes of the RCTs that evaluated PRLT. The TheraP trialists are expected to report the overall survival findings in 2022, and we intend to analyze future publications of the TheraP and VISION trials [40].

Our NMA used a frequentist model and conferred with two assumptions. As for transitivity, the participants in the RCTs were sufficiently similar [32]. As to homogeneity, patients given a specific L3 treatment in several RCTs had sufficiently homogeneous PSA responses, as shown in a forest plot [34].

Liver metastasis is an important effect modifier of overall survival after L3 treatment [42,43] but only two of the seven RCTs reported the number of patients who had liver metastases.

Some patients in our NMA had previously been treated with several series of ADT and/or been treated with several series of docetaxel. For simplification, we lumped several treatment series of ADT/ARPI as a single endocrine treatment, and lumped repeat docetaxel series as a single line of docetaxel. Thus, our NMA differed from a previous publication that counted all series of relapse treatments [44].

RCTs showed that abiraterone and enzalutamide given as an L1 treatment before docetaxel improved outcomes compared with the use of these drugs as an L2 treatment after docetaxel [45]. This explains why patients in our recent RCTs increasingly used the drugs as an L1 treatment.

L3 treatment with PRLT had a higher impact on the PSA response than on radiographic progression-free survival and overall survival. The PSA response reflects the cell killing of a treatment, whereas radiographic progression-free survival reflects the combined effect of cell killing and regrowth between the cycles/courses of treatment. Overall survival reflects the combined effect of radiographic progression-free survival and L4 treatment given after failure of L3 treatment.

For patients with mCRPC, L3 treatment is important for overall survival. L3 treatment with alternative abiraterone or enzalutamide after failure of a previous treatment with ARPIs resulted in a median overall survival of 4 months, and cabazitaxel resulted in a median overall survival of 13 to 14 months. In contrast, PLRT resulted in a median overall survival of 15.3 months [20].

The proportion of patients with severe adverse effects varied between the treatments. Most RCTs showed small proportions with severe hematological adverse effects. A study indicated that a pretreatment hematological impairment contributed to the proportion of patients with severe hematological adverse effects after L3 treatment with PRLT [46]. The TheraP trial reported several categories for the premature discontinuation of the treatments [19], so the trial had a lower rate of patients who discontinued L3 treatment due to adverse effects than other RCTs.

The TheraP and the VISION trials of PRLT as an L3 treatment used the same ^177^Lu activity for the initial cycle of PRLT and the same six-week interval between the cycles. However, cohort studies showed that patients with lymph node metastatic prostate cancer had a much longer median overall survival after treatment with PRLT [44,47] than that in the two RCTs that used PRLT for patients who had bone metastases with or without visceral metastases.

Cohort studies indicated that PRLT given as an L2 treatment before treatment with docetaxel produced a better PSA response than PRLT given as an L3 treatment after treatment with docetaxel [47,48]. Another cohort study showed that PRLT given with a four-week interval between cycles resulted in a median overall survival of >20 months [49]. A case report described the use of PRLT combined with low-dose docetaxel [50].

Ongoing trials are investigating the early use of PRLT in the treatment sequence for patients with progressing metastatic prostate cancer. A pilot study reported on the use of PRLT as an L1 treatment for patients with metastatic low-volume hormone-sensitive prostate cancer [51]. The findings motivated the hospital to commence two RCTs (NCT03828838 and NCT04443062, ClinicalTrials.gov, accessed on 30 June 2021). The UpfrontPSMA trial is examining whether giving two cycles of PRLT before L1 treatment with docetaxel increases the proportion of patients who have undetectable PSA one year after the treatment [52].

Other trials are investigating combining PRLT with established drugs [43], such as enzalutamide (the EN ZA-p trial [53]).

Previous NMAs have elucidated the efficacy of the established drugs for patients with prostate cancer [54,55,56,57,58,59,60,61,62,63,64,65,66,67,68,69,70,71,72,73,74,75], of which some NMAs have addressed RCTs of patients with mCRPC [55,64,74]. It should be noted that the 2021 EAU guidelines were based on a search of the literature from 2016 to 2019 [2]. As in our NMA referring to the CARD trial [9], the American Urology Association (AUA) guidelines from 2020, with an evidence level of B, recommended that urologists and oncologists should prefer cabazitaxel as an L3 treatment over treatment with an alternative abiraterone or enzalutamide.

In addition to prostate cancer, L3 treatments have been documented for patients with breast cancer, non-small cell lung cancer, and multiple myeloma [76,77,78].

Our NMA has limitations. PRLT is recommended only for patients with PSMA-positive metastases but the vast majority of patients who are candidates for L3 treatments have PSMA-positive metastases. ^225^Actinium-based radioligand therapy is also a promising new treatment for patients with mCRPC [13,79] but so far, its efficacy has not been documented in RCTs. Our NMA did not include treatments that were effective only for a small subgroup of patients with mCRPC, such as the PARP inhibitor olaparib for patients with BRAC mutations [80,81].

In conclusion, for patients with mCRPC, L3 treatment with PRLT is highly effective and safe.

## Figures and Tables

**Table 1:** Clinical characteristics.

Study	IXA	IXA	TRO	TRO	PROS	PROS	AFFI	AFFI	CARD	CARD	Ther	Ther	VISI	VISI
Treat	MIT	IXA	MIT	C25	C25	C20	C25	C25+C	C25	ABI	C20	PRL	BSC	PRL
Pts	41	41	377	378	602	598	318	317	129	126	101	99	280	551
Age	69	67	67	68	68	68	68	68	70	71	72	72	72	71
RP	15	16	205	198	264	272	NR	NR	NR	NR	NR	NR	82	159
EBRT	7	10	222	232	NR	NR	NR	NR	NR	NR	NR	NR	NR	NR
ADT	41	41	375	375	594	583	NR	NR	128	126	NR	NR	280	551
ABI	NR	NR	NR	NR	165	158	NR	NR	128	126	91	91	280	551
DOCE	41	41	377	378	601	584	318	317	129	126	NR	NR	280	551
PS0-1	28	28	344	350	540	539	NR	NR	123	119	96	95	258	510
Visc	NR	NR	94	94	186	187	115	108	21	25	13	7	66	112
Liver	NR	NR	NR	NR	90	94	NR	NR	NR	NR	NR	NR	12	47
PSA	141	137	128	144	172	160	NR	NR	62	61	110	94	91	91
F-up	NR	NR	13	13	NR	NR	30	30	9	9	18	18	21	21
L4	30	16	NR	NR	NR	NR	NR	NR	30	42	NR	NR	221	301

ABI: previous treatment with abiraterone or enzulatumide; ADT: androgen deprivation therapy; AFFI: AFFINITY; BSC: best supportive care; C20: cabazitaxel 20 mg/m^2^ body surface; C25: cabazitaxel 25 mg/m^2^ body surface; C25 + C: Cabazitaxel plus Custirsen; DOCE: docetaxel; EBRT: External beam radiotherapy; F-up: Median follow-up (months); IXA: Ixapiletone; L4: line 4 treatment; liver: liver metastases; MIT: mitoxantrone; NR: not reported; PRL: PSMA radioligand therapy; PROS: PROSELICA; PS0-1: performance status 0 to 1; PSA: pretreatment prostate specific antigen (ng/mL); pts: number of participants; RP: radical prostatectomy; Ther: TheraP; treat: treatment; TRO: TROPIC; Visc: visceral metastases; VISI: VISION.

**Table 2:** Outcomes.

Study	IXA	IXA	TROP	TROP	PROS	PROS	AFFI	AFFI	CARD	CARD	Ther	Ther	VISI	VISI
Treat	MIT	IXA	MIT	C25	C25	C20	C25	C25+C	C25	ABI	C20	PRLT	BSC	PRLT
PSA	20	17	18	39	43	31	NR	NR	36	14	37	66	7	46
rPFS	NR	NR	1	3	9	9	NR	NR	4	3	5	5	3	9
OS	10	10	13	15	15	13	13	14	14	11	NR	NR	11	15

Most abbreviations as in Table 1. OS: median overall survival (months); PSA: median best PSA decline ≥50% (%); rPFS: median radiographic progression-free survival (months).

**Table 3:** Ranking of treatments regarding PSA response.

Treatment	IXA	MITO	ABI/ENZA	CABA20	CABA25	PRLT	BSC
Best treatment	0.1	0.0	0.0	0.0	2.3	97.6	0.0
Worst treatment	0.0	0.0	59.1	0.0	0.0	0.0	33.1

Abbreviations as in Table 1 and Table 2.

**Table 4:** Ranking of treatments regarding radiographic progression-free survival.

Treatment	CABA20	PRLT	BSC
Best treatment	18.5	81.5	0.0
Worst treatment	0.0	0.0	100

Abbreviations as in Table 1 and Table 2.

**Table 5:** Ranking of treatments regarding overall survival.

Treatment	IXA	MITO	CABA25	CABA25 + CUST	CABA20	ABI/ENZA
Best treatment	5.4	0.0	17.9	65.1	11.4	0.2
Worst treatment	13.7	1.1	0.0	0.0	0.0	85.2

Abbreviations as in Table 1 and Table 2.
